# Long-term survival of an elderly patient with advanced gastric cancer after combination therapy: a case report and literature review

**DOI:** 10.1186/s12885-019-5683-4

**Published:** 2019-05-16

**Authors:** Qingwei Li, Xuejun Xu, Dan Su, Tianshuo Zhou, Guangyu Wang, Zhiwei Li

**Affiliations:** Department of Gastrointestinal Medical Oncology, Harbin Medical University Cancer Hospital, Harbin, 150000 China

**Keywords:** Gastric cancer, Liver metastasis, Chemotherapy, Combination therapy

## Abstract

**Background:**

Gastric cancer ranks the fifth most common cancer, and the third leading cause of cancer-related deaths worldwide. Gastric cancer with liver metastasis (GCLM) has devastating prognosis, however, optimal treatment of GCLM, especially in elderly patients, has yet to be clarified.

**Case presentation:**

A 75-year-old man was diagnosed with advanced gastric cancer (GC), presenting with acute gastrointestinal bleeding and synchronous metastatic lesion in liver. Based on multidisciplinary team (MDT)‘s decision, this patient underwent distal palliative gastrectomy with R1 margin. Histopathological diagnosis was stage IV gastric adenocarcinoma (pT3N2M1), HER2 negative. The patient was treated with chemotherapy and argon-helium cryoablation of liver and lung metastases.HER-2 gene amplification was identified in peripheral blood at later stage of therapy. The patient had been followed-up for 39 months, in sharp contrast to a median survival time of 13.8 months for majority of advanced GC.

**Conclusions:**

Palliative distal gastrectomy in combination with chemotherapy and cryoablation significantly prolongs overall survival of an elderly patient with GCLM.

**Electronic supplementary material:**

The online version of this article (10.1186/s12885-019-5683-4) contains supplementary material, which is available to authorized users.

## Background

Gastric carcinoma (GC) is the fourth most common malignancies and the third leading cause of cancer-related death worldwide, Surgical resection remains the only curative option [1]. In China, there are estimated 221,478 deaths per year due to GC, accounting for nearly half of the globally total deaths from GC [2]. Most of the patients with GC are diagnosed at advanced stages, frequently presenting invasion or metastasis [3]. Approximately, 25 and 30%, respectively, of Chinese patients have early or late (metastatic) stage GC at diagnosis. While in the United States, 36% of patients have early stage at diagnosis [4].

At present, an optimal treatment of GCLM (which is classified as M1 clinical stage) remains debated [5, 6]. Hepatic metastases from GC are considered as unresectable since these lesions present as multiple nodules, which are distributed in hepatic lobes, as well as extrahepatic organs [7, 8]. Devastating prognosis is usually expected for unresectable GC. Under this circumstance, palliative chemotherapy will be highly recommended [9]. Among patients treated with chemotherapy alone, their 5-year OS rate was only 1% (with a median survival time of 14 months). Conversion surgery may be attributed to long-term survival in selected patients.

The role of gastrectomy playing in treating metastatic GC (in the absence of urgent symptoms, such as bleeding or obstruction) is yet to be illustrated. A higher risk from surgery and longer recovery time are expected for elderly patients. Through reducing tumor volume, debulking surgery may prolong survival and/or delay the onset of life-threatening symptoms [10]. Elderly patients with GCLM are under-represented in clinical trials, with few reported studies in this setting.

Here, we have described a specific case of long-term survival after palliative distal gastrectomy combined with chemotherapy and argon-helium cryoablation of liver and lung metastases. The observed improved outcome definitely merits a prospective study to explore potential survival benefits in specifically selected patients, especially for those who urgently require palliation of serious symptoms, such as bleeding or obstruction.

## Case presentation

**Fig. 1:**
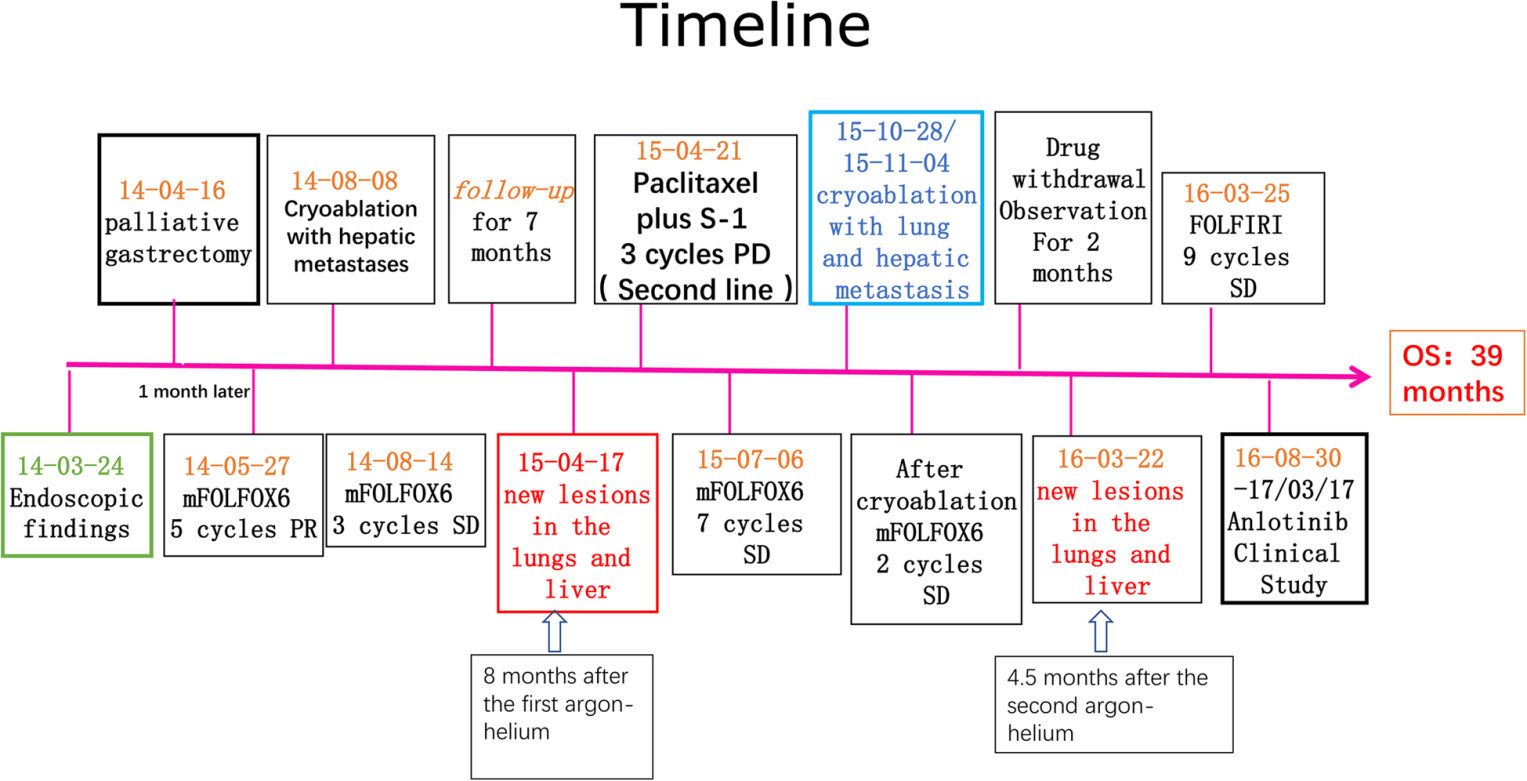
Information of this case report has been organized into a timeline

**Fig. 2:**
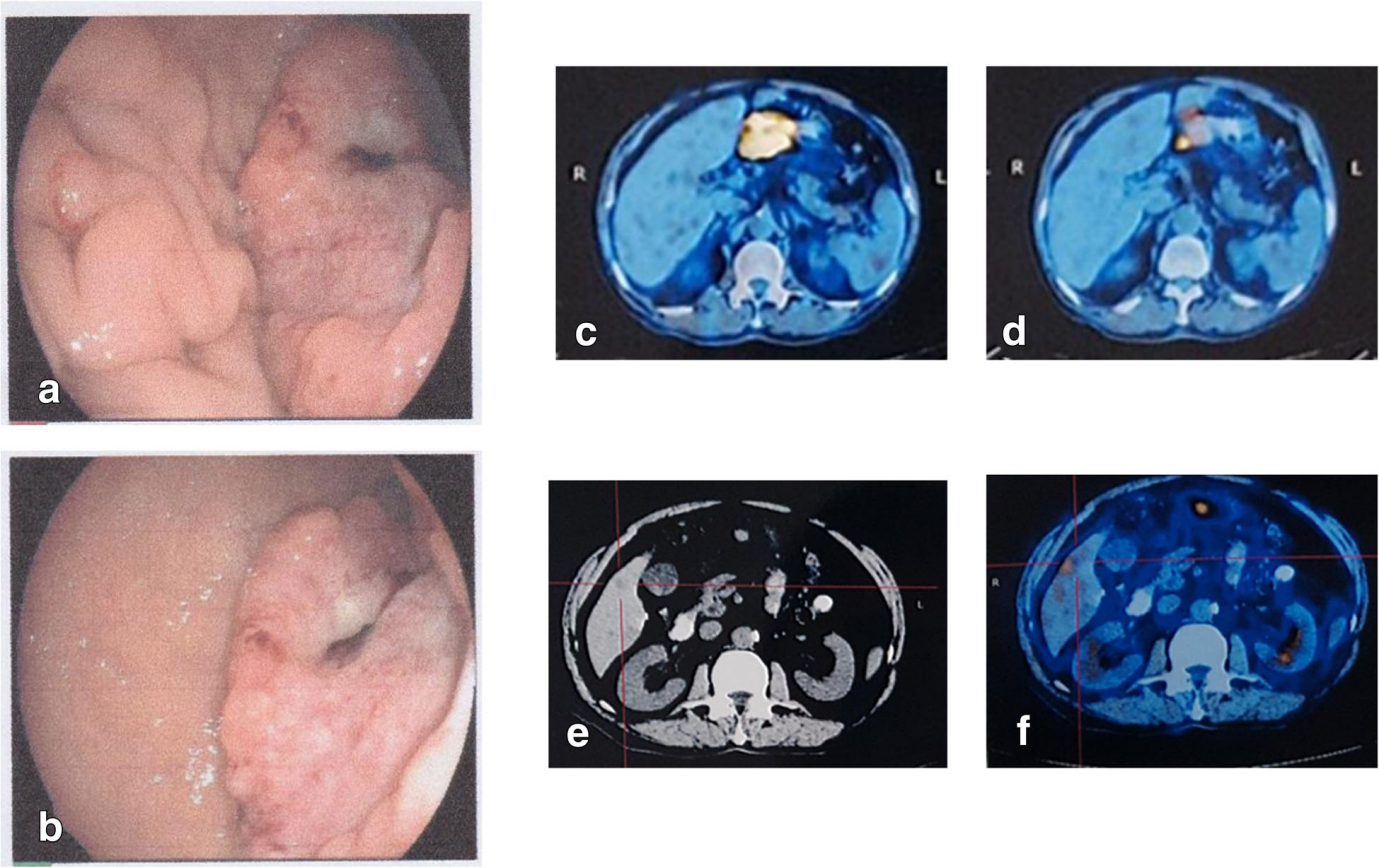
Endoscopy and PET/CT revealed GCLM. **a**. posterior wall of the antrum: worm-like erosion edges, visible bleeding from blood vessels and blood clots. **b**. small curvature of the antrum, white protrusions. **c** The lesion was located in the gastric antrum(SUVmax13.3), indicating GC. **d** Lymph node metastasis was observed below the gastric antrum (SUVmax3.3). d. Computed Tomography Image revealed a single liver metastasis, located in segments 6. **e** PET/CT identified localized radionuclide concentration (SUVmax3.5)

A 75-year-old man suffered from abdominal pain and melena. Emergency gastroscopy observed large curvature, posterior wall and small curvature of the antrum. Huge flat uplift occupying lesions were identified, with worm-like erosion edges, uneven bottom, visible bleeding from blood vessels and blood clots (Fig. 1 [green frame]; Fig. 2.a.b). An upholstery lesion (in the maximum diameter of 1.5 cm) with white protrusions was observed close to the anterior wall of small curve. Whole body fluorine-18 fluorodeoxyglucose (18F–FDG) positron emission tomography/computed tomography (PET/CT) identified a hypodense mass in segment 6 of liver. Intense 18F–FDG distribution obtained a maximum standardized uptake value (SUV) of 3.5(Fig. 2, c-e). After MDT consultation, R0 resection might be impossible to achieve. Palliative chemotherapy was relatively contraindicated due to a high risk of gastrointestinal bleeding. The patient underwent palliative gastrectomy to prevent from bleeding and perforation. No liver metastatic lesion was resected. On microscopic examination, the primary tumor was identified as a well to mixed differentiated gastric adenocarcinoma (75% papillary adenocarcinoma and 25% moderately differentiated tubular adenocarcinoma), which had invaded subserosa layer. Five of 35 lymph nodes were positive for metastases, without venous or lymphatic vasculature invasion. This GC tumor fulfilled the criteria of stage IVa (pT3N2M1), based on the American Joint Committee on Cancer (AJCC) TNM staging classification for carcinoma of the stomach (7th edition, 2012) [11]. This tumor was negative for HER2 amplification.

**Fig. 3:**
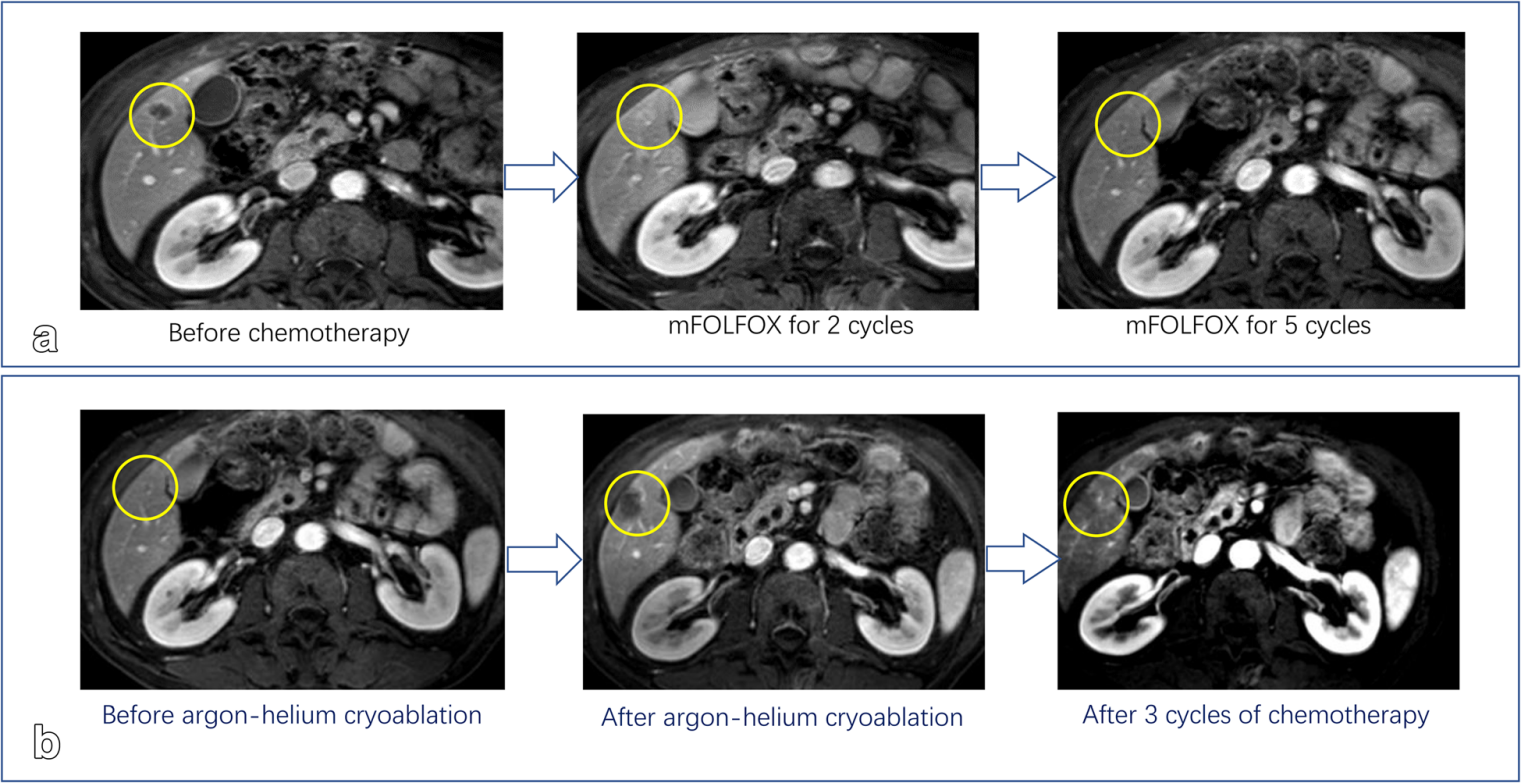
Treatment protocol after reduction surgery. **a** The patient received 5 cycles of mFOLFOX6 regimen chemotherapy. The efficacy was assessed as PR. **b** The patient received cryoablation of argon and helium with hepatic metastases, and 3 cycles of mFOLFOX6 chemotherapy. The efficacy was evaluated as SD

**Fig. 4:**
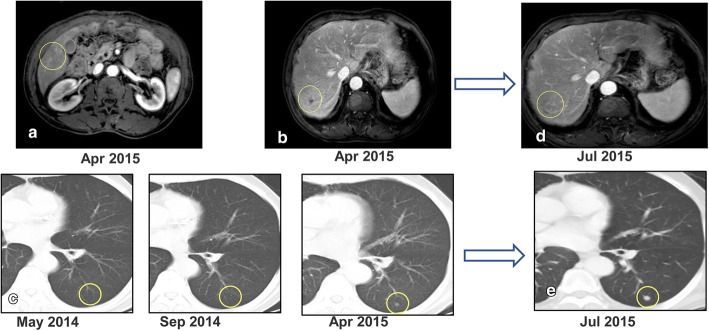
CT and MRI scans identified new lesions in the lungs and liver espectively seven months after the first-line chemotherapy and cryoablation (April 2015). **a**. lesion cryoablation was stable, arterial phase. **b**. new liver lesion. **c** New metastatic lesions in the lungs. **c** and **d**. The efficacy was evaluated as PD after Paclitaxel plus S-1 (second-line) chemotherapy since April 2015

**Fig. 5:**
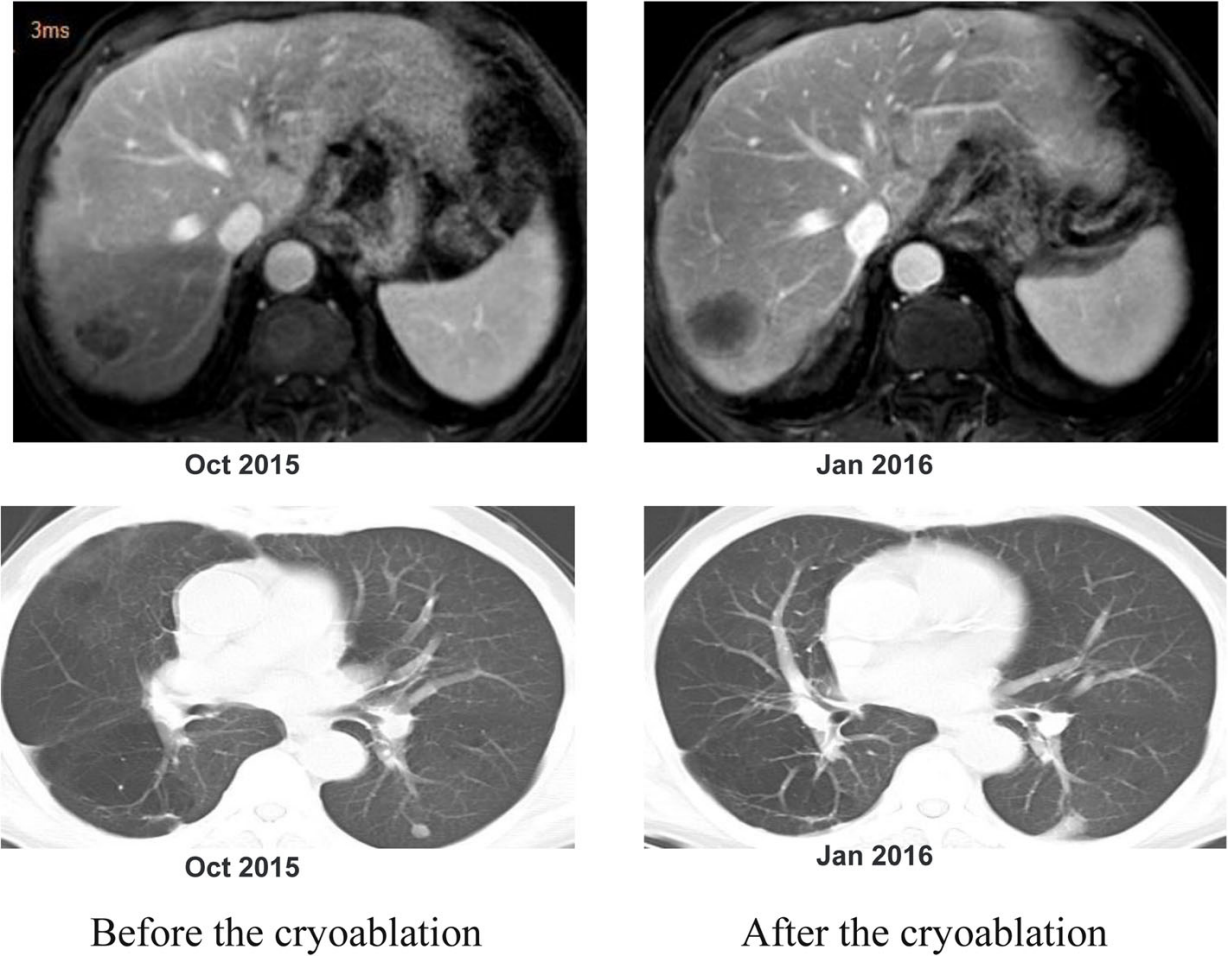
Comparison of tumor status before and after the second cryotherapy

**Fig. 6:**
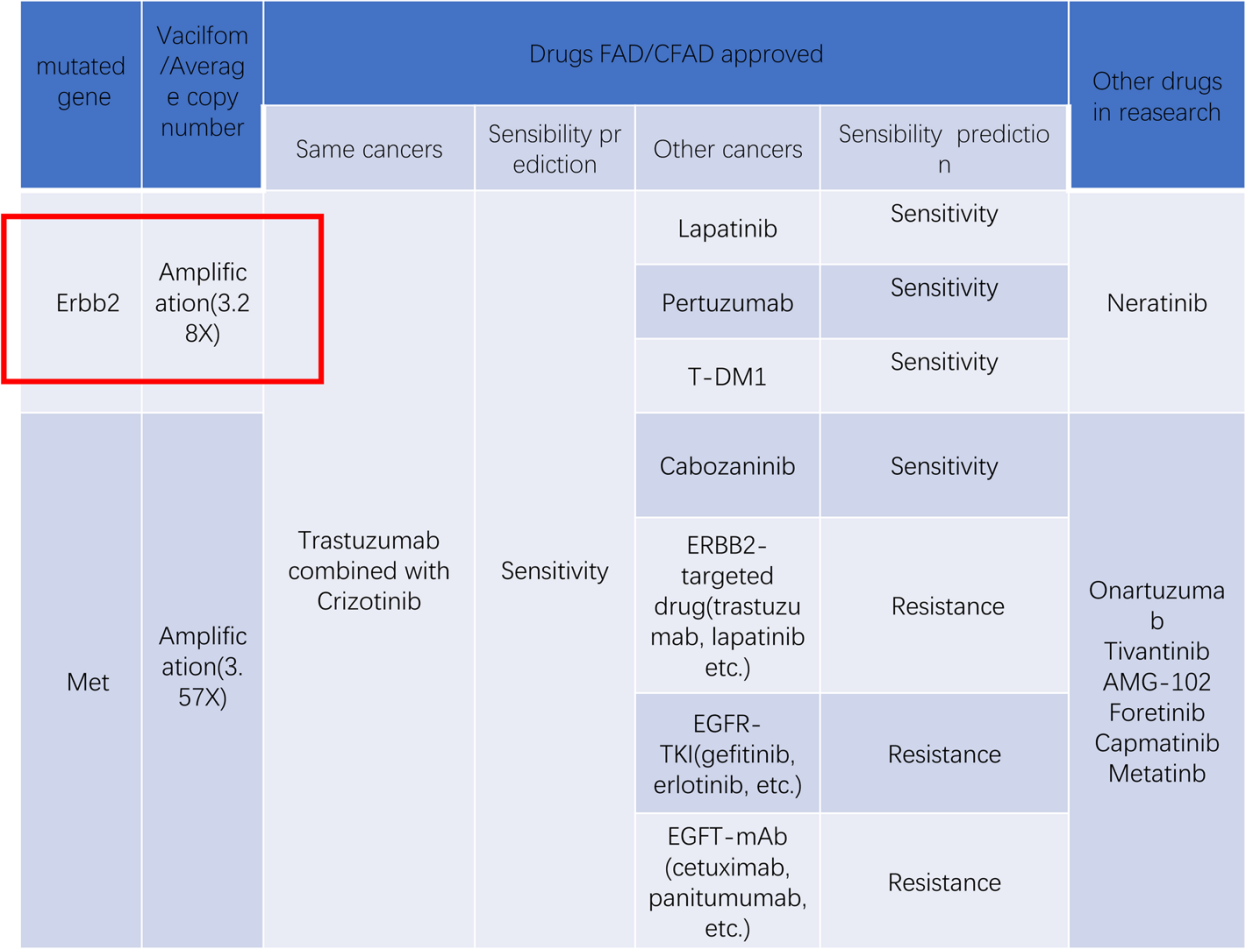
Gene sequencing using peripheral blood sample.Red box highlighted HER2 amplification, which could be treated with pertuzumab (Herceptin)

**Fig. 7:**
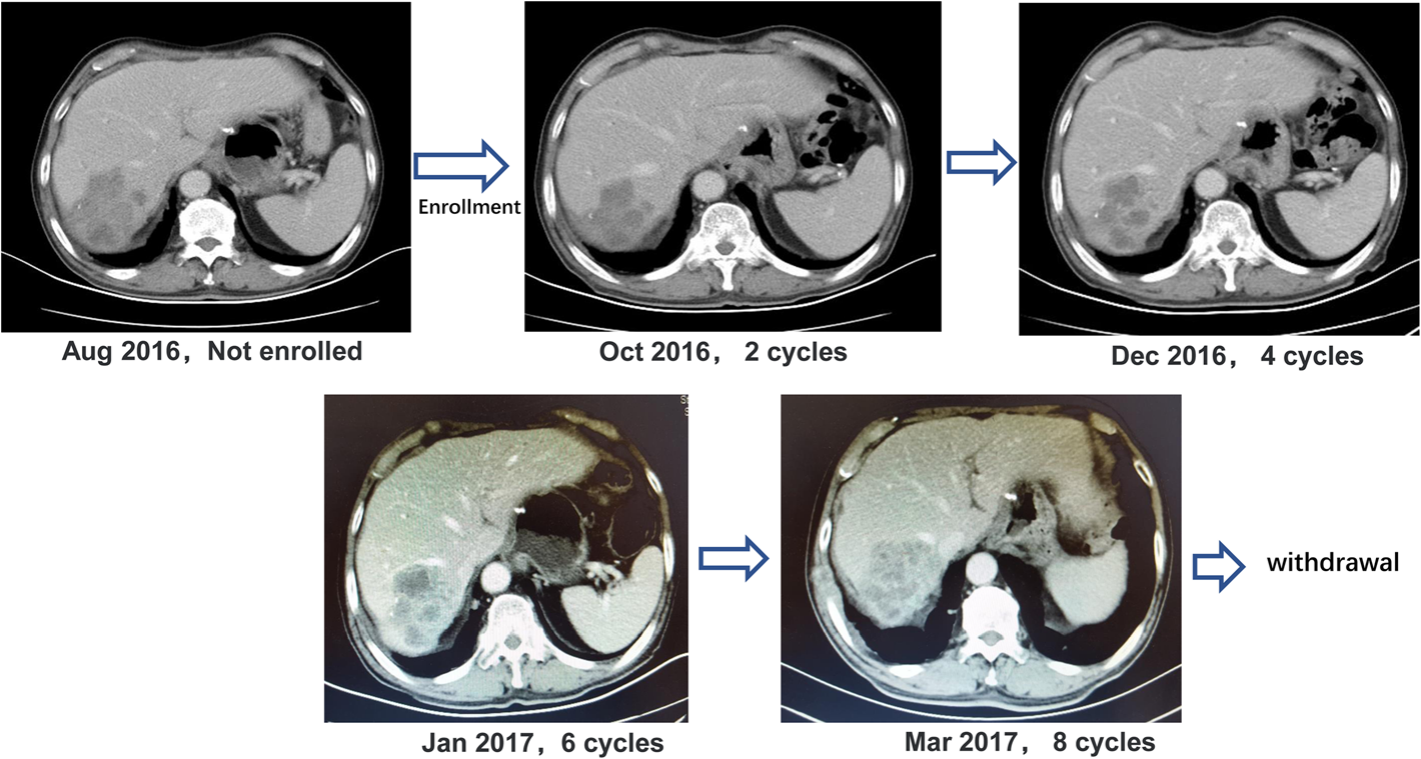
The patient was enrolled into a randomized, double-blind, placebo-controlled clinical trial of Anlotinib

In the first month post-operation, this patient was transferred to our hospital for comprehensive evaluation. He had received 5 cycles of mFOLFOX6 (5-fluorouracil/folinic acid, oxaliplatin) regimen as the first-line chemotherapy. Liver metastatic lesions were shrunk, so that the efficacy was assessed as partial response (PR) (Fig. 3a). Then the patient received cryoablation with argon and helium on hepatic metastases; subsequently received post-operational chemotherapy (mFOLFOX6 protocol) for 3 cycles. The efficacy was evaluated as stable disease (SD) (Fig. 3b). Chemotherapy was stop ped due to grade3 neutropenia. The patient was regularly followed-up afterwards. Seven months after the cessation of treatment, CT scan identified new lesions in the lungs and liver (Fig. 1 [red frame]; Fig. 4a, b, c). The patient started to receive 3 cycles of Paclitaxel plus S-1 (second-line) chemotherapy since April 2015, however, the short-term effect was assessed as progressive disease (PD)(Fig. 4d, e).Then the patient received mFOLFOX6 chemotherapy for 7 cycles, with the efficacy defined as SD. Afterwards, pulmonary and hepatic metastatic lesions were treated with cryoablation separately (Fig. 5). The patient received another 3 cycles of mFOLFOX6 regimen after cryoablation, undergoing regular observation. Six months later, new metastases were identified and the patient received FOLFIRI regimen chemotherapy for 9 cycles. The efficacy was evaluated as PD. At this point, HER-2 gene amplification was identified using peripheral blood sample (Fig. 6). During the entire treatment period, the patient’s CA19–9 and CEA were basically maintained at normal levels (Additional file 1: Figure S1). On August 30, 2016, the patient was enrolled into a randomized, double-blind, placebo-controlled trial of Anlotinib, which was designed for advanced GC as second-line therapy. After taking 6 cycles of Anlotinib (12/10 mg, d1–14, qd, po, q3w), the efficacy was evaluated as SD (Fig. 7). Treatment-related proteinuria occurred during the second cycle. After the 8th cycle, the efficacy was evaluated as PD. The patient withdrew from the clinical trial on March 17, 2017. On July 24, 2017, the patient died and his overall survival time was 39 months.

## Discussion and conclusions

GCLM refers to liver lesions originating from primary GC, which remains a major cause of GC-related deaths, with a 5-year survival rate of 0–10% in unselected cases [3]. Surgery with curative attempt is a key component for multidisciplinary approach. Hepatectomy may be performed on a small number of metastatic nodules, and not restricted to a solitary nodule, provided no other non-curable factors. Whether synchronous metastases have better prognosis than metachronous metastases remains controversial. Palliative gastrectomy could be considered for GCLM patients if they fulfill following criteria: (1) Through removing a bulky tumor, potential life-threatening symptoms such as obstruction, perforation, or bleeding maybe eliminated. (2) A decrease in tumor load may render the residual cancer cells more sensitive to adjuvant therapy. (3) Reduction in tumor volume may diminish nutrient burden on the patient exerted by the tumor. (4) As the tumor produce immunosuppressive cytokines, reducing the tumor burden may help activate anti-tumor immunologic machinery [12, 13].In this case, palliative gastrectomy contributes to prolonged survival.

Clinical benefit of resecting GCLM remains controversial, especially for elderly patients [14, 15]. Clinical outcome for hepatic resection was disappointing due to high rates of recurrence and death. Occult intrahepatic metastases at the time of surgery may lead to high incidence of intrahepatic recurrence [16]. A second hepatic resection is rarely indicated. Postoperative monitoring of liver and adjuvant chemotherapy may become a feasible strategy for improving survival [17]. In this case, chemotherapy plays a critical role in improving prognosis. Hepatic lesions were used to screen drug sensitivity vs. resistance, and mFOLFOX regimen was indicated to be more effective. Local ablation has emerged as a promising alternative or complement to resection, especially for elderly patients with GCLM. Ablative techniques include radiofrequency thermal ablation (RFA) [18, 19], microwave ablation (MWA) [20, 21], and cryoablation [22]. The average survival time of ablative combined with chemotherapy for liver metastases was 16.1 months [23]. According to the retrospective study, the 5-year survival rate after ablative treatment was not significantly different from that of hepatectomy [24]. This patient underwent 3 times of local cryotherapy. After the first cryotherapy, perioperative chemotherapy was conducted. The patient’s disease-free survival had reached 7 months. This procedure for elderly patients with GCLM is effective and safe (with low mobility), which may be performed repeatedly on an outpatient basis with a good palliative effect.

Supporting evidence from clinical trials is required to guide decision-making when treating elderly patients with GCLM. Age alone is no contraindication for resections of GI. Although no survival benefit for neoadjuvant treatment in patients over 70 years is found [25]. In this case, if the patient was not at risk of bleeding, we may prefer neoadjuvant chemotherapy. Preoperative chemotherapy significantly prolongs the survival of the esophago-gastric adenocarcinomas patient with primary resection [26]. In regards to chemotherapy for advanced/metastatic GC, the experience from general population is unlikely to be directly applicable to elderly patients [27]. It is inappropriate to estimate tolerability of elderly patients to chemotherapy based only on chronological age without considering functions of major organs, comorbidities and medical history. However, an ideal index to comprehensively assess vulnerability of aged individual has yet to be established. Therefore, more comprehensive clinical studies with larger sample sizes on elderly patients with GCLM are guaranteed to validate our findings in the near future.

This patient participated in a clinical study, which was helpful to extend overall survival. Anlotinib is an oral formulation of a small molecule inhibitor of multiple receptor tyrosine kinases, with abroad spectrum of inhibitory effects on tumor growth and angiogenesis. Anlotinib has been approved in China for treatment of patients with locally advanced or metastatic non-small cell lung cancer (NSCLC), who have undergone progression or recurrence after ≥2 lines of systemic chemotherapy. The efficacy of Anlotinib in patients with stage 3B/4 NSCLC or metastatic renal cancer (mRCC) has been demonstrated. During 6 cycles of enrollment into this clinical trial, the efficacy was evaluated as SD.

This patient was HER-2 negative based on postoperative pathological examination. However, in the latter stage of treatment, genetic test using peripheral blood identified amplification in HER-2 gene. On one hand, loss of HER2-positive status occurs after neoadjuvant therapy in patients with primary HER2-positive breast cancer [28]. On the other hand, 3.4% of breast cancer patients with HER2-negative tumors before chemotherapy changed to HER2-positive afterwards [29]. According to this case, we guess that the alteration in HER2 expression may happen in GC, which may be resulted from resistance to chemotherapy as HER2 amplification means poor prognosis.

For elderly patients with GCLM, combination therapy has efficacy. MDT consultation facilitates the evaluation of clinical stage, feasibility, risk and benefit of individual treatment modality. Palliative gastrectomy for GCLM is reasonable and safe; however, the patients must be strictly selected. Systemic chemotherapy combined with local cryoablation is an important choice for GCLM. To participate inappropriate clinical trials may be indispensable.

## Additional file

Additional file 1: **Figure S1.** Levels of change in ca 19–9 and CEA during patient treatment. During the entire treatment period, the patient’s CA19–9 and CEA were basically maintained at normal levels. A.CA19–9; B. CEA. (TIF 27264 kb)

## Abbreviations

18F–FDG: fluorine-18 fluorodeoxyglucose
CT scan: computed tomography scan
FOLFIRI: fluorouracil, leucovorin, and irinotecan
GC: gastric cancer
GCLM: gastric cancer with liver metastasis
HER-2: Receptor tyrosine-protein kinase erbB-2
MDT: multidisciplinary team
mFOLFOX6: Modified Fluorouracil, Leucovorin, and Oxaliplatin
MWA: microwave ablation
OS: overall survival
PD: progression disease
PET/CT: positron emission tomography/computed tomography
PR: partial remission
RFA: radiofrequency thermal ablation
SD: stable disease
SUV: standardized uptake value
