# Supplementary figures and images for: Long-term survival of an elderly patient with advanced gastric cancer after combination therapy: a case report and literature review
Source: BMC Cancer. 2019 May 16;19:459. doi: 10.1186/s12885-019-5683-4 (PMC6524267; doi:10.1186/s12885-019-5683-4)

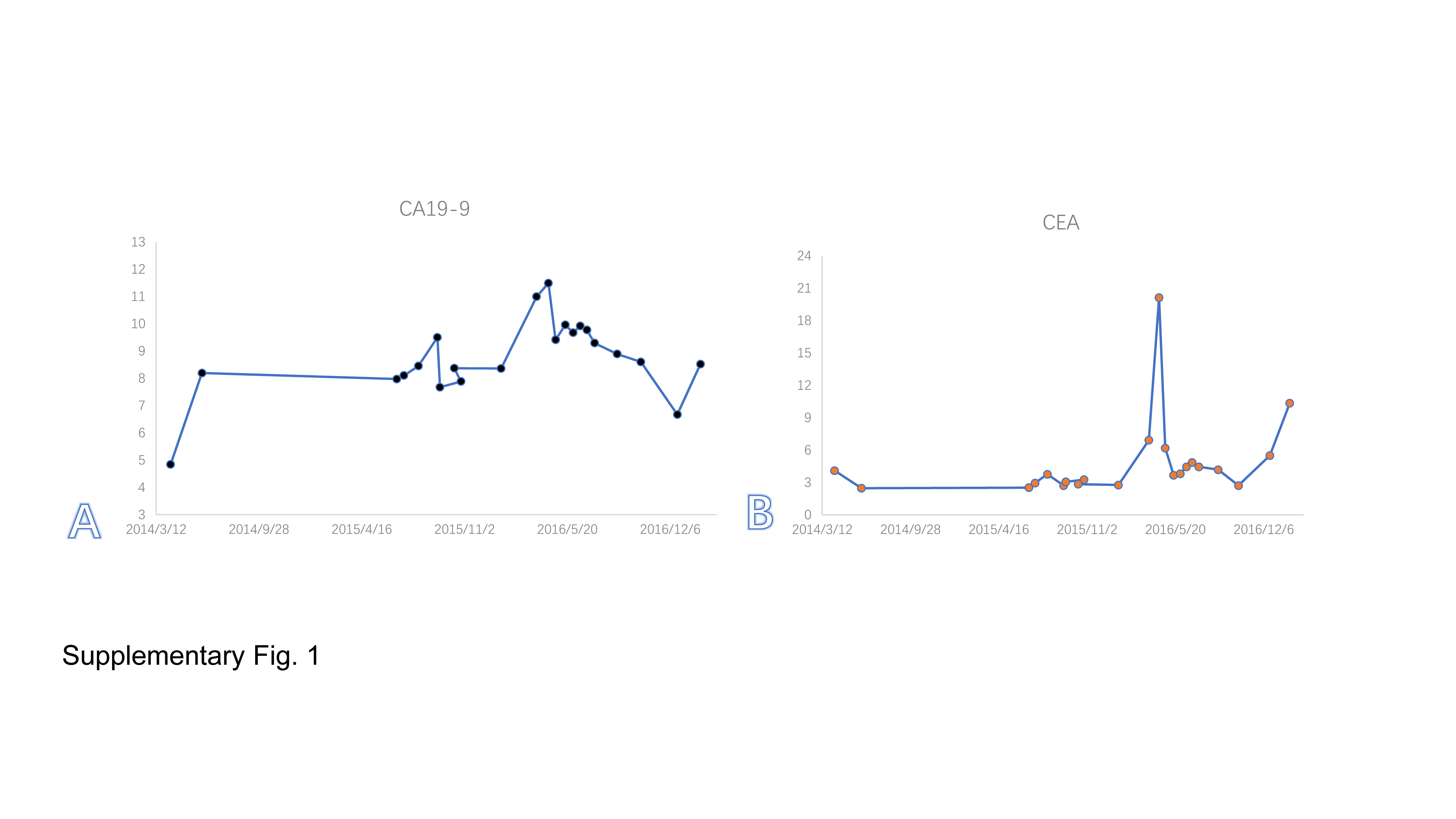

Supplement: Supplementary file 1 — Figure S1. Levels of change in ca 19–9 and CEA during patient treatment. During the entire treatment period, the patient’s CA19–9 and CEA were basically maintained at normal levels. A.CA19–9; B. CEA. (TIF 27264 kb) [file 12885_2019_5683_MOESM1_ESM.tif]
